# Audition and vision share spatial attentional resources, yet attentional load does not disrupt audiovisual integration

**DOI:** 10.3389/fpsyg.2015.01084

**Published:** 2015-07-29

**Authors:** Basil Wahn, Peter König

**Affiliations:** ^1^Neurobiopsychology, Institute of Cognitive Science, Universität OsnabrückOsnabrück, Germany; ^2^Department of Neurophysiology and Pathophysiology, Center of Experimental Medicine, University Medical Center Hamburg-EppendorfHamburg, Germany

**Keywords:** attentional load, multisensory integration, auditory display, vision, audition, attentional resources, multiple object tracking

## Abstract

Humans continuously receive and integrate information from several sensory modalities. However, attentional resources limit the amount of information that can be processed. It is not yet clear how attentional resources and multisensory processing are interrelated. Specifically, the following questions arise: (1) Are there distinct spatial attentional resources for each sensory modality? and (2) Does attentional load affect multisensory integration? We investigated these questions using a dual task paradigm: participants performed two spatial tasks (a multiple object tracking task and a localization task), either separately (single task condition) or simultaneously (dual task condition). In the multiple object tracking task, participants visually tracked a small subset of several randomly moving objects. In the localization task, participants received either visual, auditory, or redundant visual and auditory location cues. In the dual task condition, we found a substantial decrease in participants' performance relative to the results of the single task condition. Importantly, participants performed equally well in the dual task condition regardless of the location cues' modality. This result suggests that having spatial information coming from different modalities does not facilitate performance, thereby indicating shared spatial attentional resources for the auditory and visual modality. Furthermore, we found that participants integrated redundant multisensory information similarly even when they experienced additional attentional load in the dual task condition. Overall, findings suggest that (1) visual and auditory spatial attentional resources are shared and that (2) audiovisual integration of spatial information occurs in an pre-attentive processing stage.

## 1. Introduction

From all our senses, we continuously receive far more information than can be effectively processed. Via a process called “attention” (James, 1890; Chun et al., 2011), we select information that is relevant for our current situation. In the present study, we investigate the relation between attention and multisensory processes. In particular, we investigate whether attentional processing draws from separate pools of attentional resources for each sensory modality and to what extent attentional resources interact with multisensory integration processes.

Regarding the first question, it has been shown that the amount of information that can be attended at once is limited (Marois and Ivanoff, 2005; Alvarez and Franconeri, 2007). Attentional limitations were found in hearing (Tremblay et al., 2005), vision (Potter et al., 1998), and haptics (Hillstrom et al., 2002). The question of whether attentional limitations are specific to each sensory modality or whether there is a common pool of attentional resources for all sensory modalities is a matter of ongoing debate (for support for distinct attentional resources see: Duncan et al., 1997; Potter et al., 1998; Soto-Faraco and Spence, 2002; Alais et al., 2006; Hein et al., 2006; Talsma et al., 2006; van der Burg et al., 2007; for support for a common pool of resources see: Jolicoeur, 1999; Arnell and Larson, 2002; Soto-Faraco et al., 2002; Arnell and Jenkins, 2004). In particular, if humans have separate attentional resources for each sensory modality, the total amount of information that can be attended to would be larger if the received information would be distributed across several sensory modalities rather than received only via one sensory modality.

Earlier studies proposed that whether humans have separate attentional resources or one common pool of resources depends on the type of task (Bonnel and Prinzmetal, 1998; Potter et al., 1998; Chan and Newell, 2008; Arrighi et al., 2011). In particular, Arrighi et al. (2011) argued that when humans carry out tasks that require attention over longer periods of time (i.e., sustained attention) rather than for a brief time, they employ separate attentional resources from each sensory modality as opposed to a common pool of attentional resources. In their study, participants performed a multiple object tracking (“MOT”) task (Pylyshyn and Storm, 1988) while concurrently performing either a visual or auditory discrimination task. In a MOT task, participants visually track a subset of objects (“targets”) among other randomly moving objects (“distractors”) for several seconds—a task requiring visual spatial attention over extended periods of time. When participants performed the MOT task and the visual discrimination task at the same time, Arrighi et al. (2011) found strong within-modality interference. However, when participants performed the MOT task and the auditory discrimination task at the same time, Arrighi et al. (2011) found little cross-modality interference. These findings suggest that there are separate attentional resources for the visual and auditory modalities in tasks that require sustained attention.

Notably, in Arrighi et al. (2011), participants performed a discrimination task and a spatial task at the same time. These findings left an open question whether humans employ separate attentional resources only when simultaneously performing a spatial task and a discrimination task (both requiring sustained attention) or whether they also employ separate attentional resources when performing two spatial tasks that require sustained attention simultaneously. For the case of visual and tactile attentional resources, we addressed this question in a previous study (Wahn and König, 2015). Participants performed a MOT task while simultaneously performing a localization (“LOC”) task in which they either received visual, tactile, or redundant visual and tactile location cues. We reasoned that if two spatial tasks performed in separate sensory modalities draw from at least partially separate pools of attentional resources, then interference between these two spatial tasks should be less in comparison to the interference observed when two spatial tasks are carried out within the same sensory modality. However, findings revealed that, while there was substantial interference between tasks, the amount of interference did not differ between conditions in which tasks were performed in separate sensory modalities (i.e., haptics and vision) in comparison to a purely visual condition. These results indicate shared attentional resources for the visual and haptic modality when two spatial tasks are performed. Taken together, these findings suggest that distinct attentional resources for the sensory modalities are employed during simultaneous performance of a discrimination task and a spatial task, whereas a common pool of attentional resources is used during simultaneous performance of two spatial tasks (for a similar claim that spatial attention acts supramodally, see LaBerge, 1995; Chan and Newell, 2008). Moreover, this summary of results from previous studies is supported in terms of neuronal populations that potentially overlap in processing when two spatial tasks are performed in separate sensory modalities as both recruit neural substrates from a supramodal “where” pathway residing in the parietal lobe (see Maeder et al., 2001; Reed et al., 2005; Ahveninen et al., 2006 for neural substrates for the “where” and “what” pathway). In contrast, the recruited neural substrates when performing a discrimination task overlap less with those recruited in a spatial task, which could potentially explain why distinct attentional resources are found when a spatial and discrimination task are performed simultaneously (Arrighi et al., 2011).

An important difference between our previous study and the study by Arrighi et al. (2011) are the sensory modalities in which participants carried out the tasks (i.e., vision and audition in their study; vision and haptics in our previous study). Therefore, in order to test our hypothesis that spatial attention acts supramodally, the present study investigates whether humans also employ a common pool of attentional resources when performing a visual spatial task in combination with an auditory spatial task. For this purpose, we modified the experimental paradigm of the previous study and used auditory location cues instead of tactile location cues. Specifically, participants performed a MOT task while simultaneously performing a LOC task in which they either received visual, auditory, or redundant visual and auditory location cues. We hypothesized that if there are separate spatial attentional resources for the auditory and visual modalities, participants' tracking performance in the visual MOT task and localization performance in the LOC task should be better when receiving auditory or redundant visual and auditory location cues than when receiving visual location cues in the LOC task. Conversely, if spatial attentional resources are shared between visual and auditory modalities (i.e., there is only a common pool of attentional resources), no differences in performance are expected.

In addition to investigating the question of separate attentional resources for the sensory modalities, we also investigated the second question: to what extent attentional processes interact with multisensory integration processes. Previous studies showed that the “ventriloquist effect” is not influenced by attentional processes, indicating that multisensory integration occurs prior to attentional processing (Bertelson et al., 2000; Vroomen et al., 2001). Other instances of audiovisual integration, such as the “McGurk effect” (McGurk and MacDonald, 1976) were shown to occur pre-attentively (Soto-Faraco et al., 2004); as another example, also see the “pip and pop effect” (van der Burg et al., 2008). However, other studies found that selective attention positively modulated multisensory integration processes if stimuli from both sensory modalities were fully attended (Talsma and Woldorff, 2005; Talsma et al., 2007) or attenuated multisensory integration processes if only one sensory channel was attended (Mozolic et al., 2008), arguing against a purely pre-attentive account of multisensory integration. Furthermore, Alsius et al. (2005) found that increasing the attentional load via a secondary visual or auditory task severely affected audiovisual integration, suggesting that attentional processes can negatively affect multisensory integration (see also Alsius et al., 2007 for a study in which attention directed to the tactile modality weakens audiovisual integration). Koelewijn et al. (2010) suggested that multisensory integration processes do rely on attentional processes, explaining why high attentional load interferes with and selective attention influences multisensory integration.

However, most of these studies focused on the integration of auditory and visual information during the perception of speech (Navarra et al., 2010); but also see (Vroomen et al., 2001) for a study about the integration of emotional auditory and visual information. Thus, it remains unclear whether attentional load disrupts audiovisual integration for non-speech stimuli. In order to address this question, we investigated whether the integration of visual and auditory cues in a LOC task is disrupted by a high attentional load. To this end, we tested whether multisensory cue integration (Ernst and Bülthoff, 2004; Ernst, 2006) occurs in conditions of high attentional load (i.e., when the MOT task was performed simultaneously with the LOC task), and low attentional load (i.e., when only the LOC was performed). In particular, we tested whether, irrespective of the attentional load, redundant location cues in the visual and auditory modality lead to better and less variable location estimates in comparison to estimates obtained from receiving only unimodal location cues. If high attentional load does disrupt multisensory integration (as shown for instance in Alsius et al., 2005), people should no longer be able to integrate redundant information from the auditory and visual modalities in the condition of high attentional load.

## 2. Methods

### 2.1. Methods of data acquisition

#### 2.1.1. Participants

We recruited nine students (six female, *M* = 25.22 years, *SD* = 2.54 years) as participants at the University of Osnabrück. All participants had normal vision and normal hearing. We admitted only students who did not play video games on a regular basis to the study, as action video game experience can lead to considerably higher tracking performances in MOT tasks (Green and Bavelier, 2006). The ethics committee of the University Osnabrück approved the study, and all participants were informed about their rights and signed a written consent form. All participants received a monetary reward or course credits for participation.

#### 2.1.2. Experimental setup

Participants wore headphones (Sony MDR-1RNC) and sat in a dark room at a distance of 90 cm in front of a computer screen (BenQ XL2420T, resolution 1920 × 1080, 120 Hz), subtending a visual field of 32.87 × 18.49 visual degrees. We recorded eye movements with a remote eyetracking system (Eyelink 1000, monocular pupil tracking, 500 Hz sampling rate). To calibrate eye position, we used a five-point grid and repeated the calibration procedure until the maximum error was below 0.7°.

#### 2.1.3. Experimental conditions and experimental procedure

**Figure 1:**
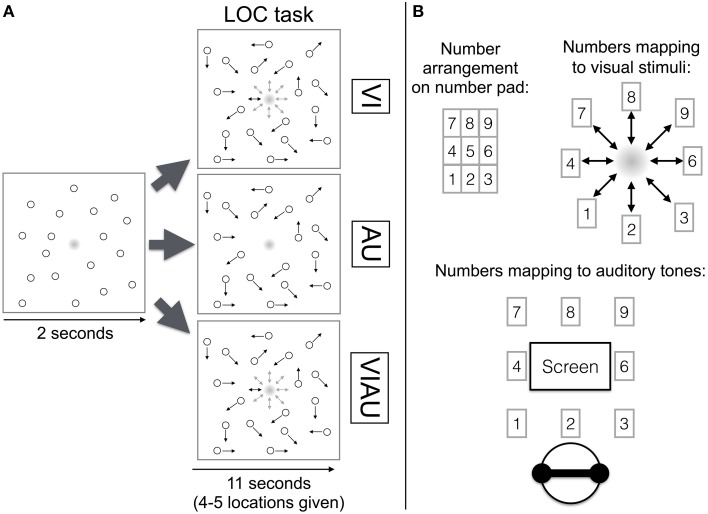
**(A)** Localization (LOC) task overview. The top row depicts the VI condition (in which visual location cues were received), the middle row the AU condition (in which auditory location cues were received) and the bottom row the VIAU condition (in which redundant visual and auditory location cues were received). **(B)** Mapping of number pad (top left) to visual stimuli on the screen (top right) and the auditory spatial cues (bottom). Arrows indicate the objects' current movement direction. This figure was adapted from our previous study (Wahn and König, 2015) with permission of Koninklijke Brill NV.

In the experiment, participants performed either a MOT task, a LOC task, or both tasks at the same time. In the LOC task, participants either had to identify the location toward which a visual, auditory, or redundant visual and auditory location cues moved. In particular, in the visual LOC task (“VI”), the participants' task was to indicate the location to which a dot (2.4 visual degrees wide) in the center of the screen moved using the corresponding key on the keyboard's number pad. The dot was a gradient (i.e., with increasing eccentricity, the color gradually changed from black to white—1 pixel change in eccentricity equaled a change in the RGB code by one unit, see Figure 1B).

During each trial, the dot moved four to five times toward one out of eight possible locations (movement length 0.03 visual degrees), remained there for 600 ms, and then returned to the center of the screen (see top row in Figure 1A). The apparent motion was created by no longer showing the dot in the center of the screen and displaying it in one of the eight positions. When there was no movement toward a location, the dot remained continuously visible in the center of the screen. The participant was allowed to give her response once she was able to identify the movement direction. The spatial arrangement of the keys on the number pad matched the eight possible locations to which the visual stimulus could move (see Figure 1B). For instance, if participants saw a movement toward the bottom right, they had to press the “3” on the number pad. The location was chosen randomly out of the eight possible locations, and onsets of these movements were jittered within a time window of 0.6 or 1 s. The minimum time between onsets was 1.5 s. Participants were asked to indicate the location toward which the dot moved but not the central location, toward which the dot always moved back. In each trial, participants performed the LOC task for 11 s, and the trial ended after this period. Within this period, participants were instructed to always fixate on the center of the screen and ignore the motions of objects that moved across the screen.

Analogously, in the auditory LOC task (“AU”), an auditory location cue originating from the center of the screen moved to one of eight auditory spatial cues surrounding the central auditory cue. The apparent motion was created by no longer playing the central sound and playing one of the adjacent sounds instead. The participants' task was to indicate the location to which the auditory cue moved using the number pad (see middle row of Figure 1A). The mapping between the auditory cues and keys on the number pad matched with regards to their spatial arrangement. For instance, the key “9” corresponded to the right auditory cue in the top row (see Figure 1B). When there was no movement toward a location, the central tone was played, in analogy to always seeing the dot in the center of the screen during the VI condition when it did not move to a location.

As auditory location cues, nine gray noise sounds (stereo sound, sampling frequency 44,100 Hz, 32 bit IEEE float format, SPL: 20.1 dBA) were created using the Wave Arts plugin Panorama 5 and Adobe Audition. Each auditory cue was simulated to be perceived as originating from a different spatial location in front of the participant. The spatial locations were composed of unique combinations of one of three horizontal angles (−90°, 0°, 90°) and one of three vertical angles (−90°, 0°, 90°). Within the Panorama 5 plugin, default options were used (stereo width: 30°, direct gain: 0 db, direct slope: −3 db, mode: headphones). For the head-related transfer function, the generic 'Human' head-related transfer function (filter length: 128 points) was selected. Reflection and reverb options were disabled.

When redundant visual and auditory location cues were received (“VIAU”), the dot and the tone moved to matching locations, and the participants' task was to indicate the location using the number pad (see bottom row of Figure 1A).

**Figure 2:**
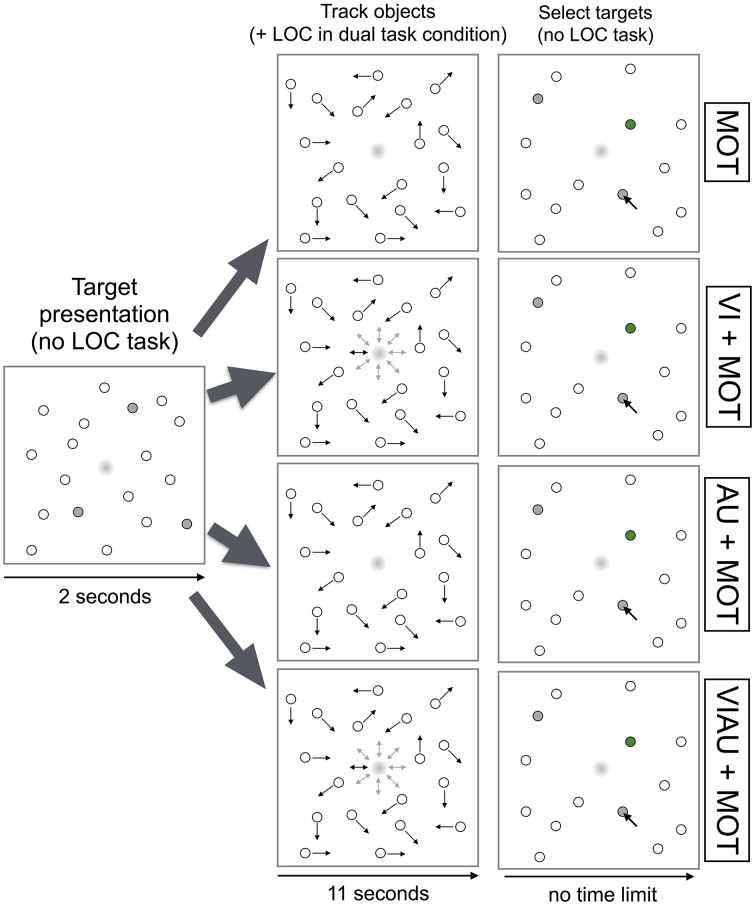
**Multiple object tracking (MOT) task overview**. Trial logic shown for the MOT task (top row), for performing the MOT task while either receiving the visual location cues (VI+MOT, second row), the auditory location cues (AU+MOT, third row) or the redundant visual and auditory location cues (VIAU+MOT, fourth row) in the localization (LOC) task. Arrows indicate the current movement direction of the objects. This figure was adapted from our previous study (Wahn and König, 2015) with permission of Koninklijke Brill NV.

In the MOT task, we instructed participants to track a subset of three randomly chosen objects (“targets”) among eighteen randomly moving objects for a total of 11 s. Before the objects (1.06 visual degrees wide) started to move, targets turned gray for a duration of 2 s and then became indistinguishable from the other objects. Then, objects moved for 11 s. During object motion, objects repelled each other and bounced off borders of the screen. Each objects' movement direction and speed [mean speed 2.57 visual degrees per second (minimum 1.71, maximum 3.42)] was randomly chosen, with a probability of 1% in each frame (the experiment was run at a 100 Hz refresh rate). When objects stopped moving, participants were instructed to select the target objects using the mouse (see Figure 2, top row). After selection of objects was complete, correctly selected objects were marked in green. In what will be referred to as “single task condition,” participants either performed one of the above described modality-specific versions of the LOC task (i.e., VI, AU, or VIAU) or the MOT task; i.e., they only performed a single task at the same time.

In the “dual task condition,” participants performed the MOT task in combination with the LOC task; they received visual (“VI+MOT”), auditory (“AU+MOT”), or redundant visual and auditory (“VIAU+MOT”) location cues. Specifically, participants first saw a screen in which objects did not move, and targets were indicated in gray for 2 s. Then, objects moved for 11 s, and participants were additionally required to perform the LOC task. Using the number pad, participants were instructed to choose the locations indicated either by the dot in the center of the screen (“VI+MOT”), by the auditory cues (“AU+MOT”), or by the dot and the auditory cues (“VIAU+MOT”)—see Figure 2, the second, third, and fourth rows, respectively. When objects stopped moving, participants were instructed to select the targets, and they no longer had to perform the LOC task.

Note, in order to keep the perceptual load constant, participants always saw eighteen randomly moving objects in each experimental condition. In addition, while tracking these objects and/or performing the LOC task, participants were instructed to always fixate on the center of the screen.

The experiment was divided into 21 blocks each consisting of ten trials, presented in a pseudorandomized order. In one block, participants always performed the same condition, which was indicated at the beginning of each block. In conditions in which a localization task was performed and given that in every trial four to five location cues were indicated, each direction for a location cue was indicated approximately seventeen times. Each set of seven blocks included all seven conditions (VI, AU, VIAU, MOT, VI+MOT, AU+MOT, VIAU+MOT). Repetition of a condition in consecutive blocks was avoided. After every seventh block, we offered participants an optional break. The entire experiment took about 2 h. We programmed the experiment and performed data extraction with Python, using the Pygame library.

### 2.2. Methods of data analysis

We excluded trials in which a participant's gaze deviated from the center by more than two visual degrees on average from the analysis (total of 2.18% trials excluded, *M* = 2.56 visual degrees, *SD* = 0.70). For each dependent variable, we regarded trials values below or above three times the interquartile range (relative to the median) as outliers and removed them per individual for each condition. We averaged all remaining trials for each participant and for each condition.

In order to test our hypotheses, we computed linear mixed models with predictors always representing planned comparisons between conditions. For this purpose, we used either a “dummy” or a “simple” coding scheme (Bruin, 2014). For the estimation of the predictors' coefficients, a maximum likelihood estimation was used, as it leads to better approximations of fixed effects than a restricted maximum likelihood estimation does (Twisk, 2006). Significance of estimated fixed effects was evaluated using 95% confidence limits. The fixed effects coefficients displayed in tables are unstandardized. For each linear mixed model, we modeled individual intercepts for each participant in order to account for the dependence between measurements across conditions (Twisk, 2006). We checked the assumptions of linearity and homoskedasticity by visual inspection of the fitted values plotted against the residuals. We assessed the assumption of normality by visual inspection of histograms of the residuals and normal Q–Q plots and by performing a Shapiro–Wilk-test (alpha = 0.1) on these residuals. In cases of violations of normality, we bootstrapped 95% confidence limits to evaluate the significance of estimated coefficients.

We used custom R scripts for all analyses. We generated tables using “texreg” (Leifeld, 2013), created graphics using “ggplot2” (Wickham, 2009), and calculated linear mixed models analyses using “lme4” (Bates et al., 2014).

## 3. Results

### 3.1. Do audition and vision share spatial attentional resources?

**Figure 3:**
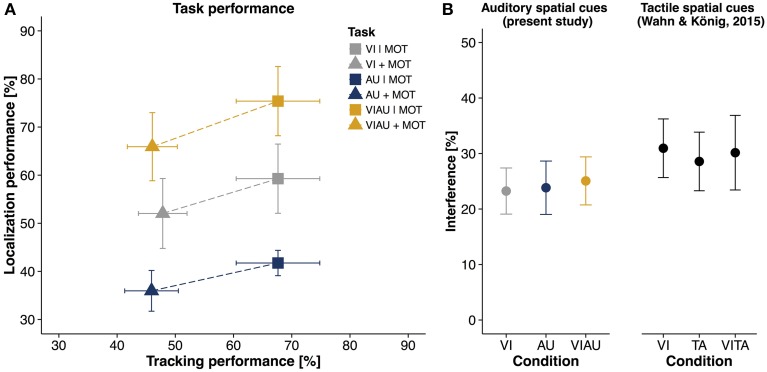
**Results of multiple object tracking (MOT) task and localization (LOC) task**. Percentage correct in MOT task (abscissa) plotted against percentage correct in LOC task (ordinate) for single task conditions of the two tasks (VI | MOT, AU | MOT, and VIAU | MOT) and dual task conditions (MOT+VI, MOT+AU, and MOT+VIAU). Dotted lines indicate the Euclidean distance between single and dual task conditions and represent an overall measure of interference; and **(B)** Interference [%] between the MOT and LOC task for each type of location cue (measured as Euclidean distance between single and dual task conditions) for the present study (left) and for the previous study (right), in which tactile instead of auditory spatial cues were received (Wahn and König, 2015), using the same interference measure. Error bars in all panels are SEM. Panel **(B)** was adapted from our previous study (Wahn and König, 2015) with permission of Koninklijke Brill NV.

Figure 3A shows a descriptive overview of the performance for the MOT task (on the abscissa) and the LOC task (ordinate), respectively. The two tasks interfered with each other irrespective of the sensory modality in which locations cues were received. To address the question of whether there are separate attentional resources for the visual and auditory modalities, it does not suffice to look at performances of each task separately. Therefore, in order to have an overall score of interference between tasks, we computed the Euclidean distance in performances between single task conditions and dual task conditions for each condition separately (Euclidean distance indicated as dashed lines in Figure 3A). Figure 3B (left panel) shows the mean Euclidean distance for the VI, AU, and VIAU conditions, respectively. It can be seen that the amount of interference in each condition is about equal in percentage, indicating that there is only one pool of attentional resources instead of separate attentional resources for each sensory modality. We tested this observation by comparing the VI condition and the AU and VIAU conditions, using a linear mixed model. For this model, we coded the predictor condition (with levels VI, AU, and VIAU) using a simple coding scheme with the VI condition as a reference group. With the simple coding scheme, the model's intercept represents the grand average over all conditions and thereby an overall score of interference between tasks. The coefficients in the model represent the comparisons between the VI condition with the AU and VIAU condition, respectively. We found a significant intercept that was also large in magnitude compared to zero (about 24%), indicating that the dual task conditions led to a considerable decrease in performance relative to the single task conditions. However, we did not find any significant differences between conditions (see first column of Table 1), indicating that there is a common pool of spatial attentional resources for the auditory and visual modalities. Moreover, these results closely match the results of our previous study (Wahn and König, 2015), in which we used tactile spatial cues instead of auditory spatial cues (for comparison, see the right panel of Figure 3B) and found evidence for shared spatial attentional resources for the tactile and visual modalities.

**Table 1:** **Linear mixed model results**.

	**Interference [%]**	**Residuals**
Intercept	24.05^*^	0.00
	[15.27; 32.82]	[−1.41; 1.41]
VI vs. AU	0.60	1.85
	[−3.74; 4.93]	[−1.61; 5.31]
VI vs. VIAU	1.83	0.67
	[−2.51; 6.16]	[−2.79; 4.13]
Log likelihood	−92.48	−72.99
Participants	9	9

*Dependent variables are “Interference [%]” between LOC and MOT task (first column) and “Interference [%]” controlled for differences in localization performance (second column). Unstandardized coefficient estimates of the differences between conditions are displayed*.

* *0 outside 95% confidence interval*.

In addition, in order to control for differences in localization difficulty in the auditory and visual modality, we computed a linear mixed model in which the localization performances in the single task conditions was used as a predictor for the interference measure. We found a trend toward significance for the relation between the localization performance and the interference measure (95%-*CI* [−0.04; 0.18], log likelihood = −92.02), indicating that the localization performance predicts the interference to some extent. Using the residuals from this model for further analysis, we regressed out all the variance that is explained by the localization performance. With the residuals, we computed the same linear mixed model as used for the interference measure and again found no significant difference between conditions (see the second column of Table 1), suggesting shared spatial attentional resources for the visual and auditory modalities.

We also suspected that there could be an asymmetry in localization performance of the auditory stimuli between the horizontal and vertical dimensions. In an extreme case, it could be that participants perfectly identified the location of the cue in one dimension while only guessing it in the other dimension. To rule out this possibility, we tested whether participants identified the given location cues in the auditory LOC task with a performance above chance (33%) only within the horizontal and vertical dimension, respectively, using a one-sample *t*-test. We found that participants indeed identified the location cues with a performance above chance for each dimension [vertical: *t*_(8)_ = 6.49, *p* < 0.001, 95%-*CI* [0.45,0.59]; horizontal: *t*_(8)_ = 13.75, *p* < 0.00001, 95%-*CI* [0.75,0.92]], ruling out the possibility that participants guessed the location of the cue in either of the dimensions.

### 3.2. Is audiovisual integration disrupted by attentional load?

In order to test whether the multisensory integration of localization cues received from the auditory and visual modalities is disrupted by attentional load, we verified whether the predictions of cue integration are fulfilled in the VIAU condition and in the MOT+VIAU condition. Given that the cues in each sensory modality give redundant information, cue integration predicts a better and also more reliable estimate of a parameter (Ernst and Bülthoff, 2004; Ernst, 2006). For our paradigm, this means that participants should be more accurate (i.e., commit fewer errors) in estimating the locations in the LOC task in the VIAU condition compared to the AU and VI condition. Furthermore, the standard deviations of participants' location estimates should be lower in the VIAU condition than in the AU and VI conditions, indicating more reliable estimates of the locations in the VIAU condition. Conversely, if multisensory integration is disrupted by attentional load, the predictions of cue integration should not be fulfilled. In particular, participants should not be more accurate and more reliable in estimating the locations in the VIAU condition compared to doing so in the AU and VI conditions.

**Figure 4:**
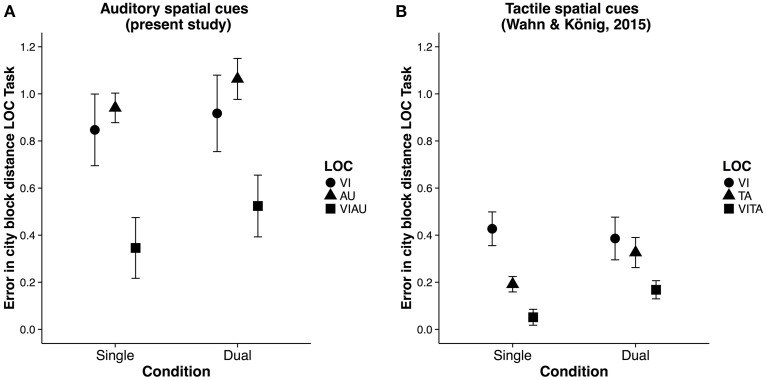
**Results of the localization (LOC) task**. **(A)** Error (in city block distance) in LOC task for each type of location cue [visual (VI), auditory (AU) and redundant auditory and visual location cue (VIAU)], separately for single and dual task conditions (present study). **(B)** Error (in city block distance) in LOC task for each type of location cue: visual (VI), tactile (TA) and redundant tactile and visual location cue (VITA), separately for single and dual task conditions from the previous study (Wahn and König, 2015). Error bars in all panels are SEM. Panel **(B)** was reproduced from our previous study (Wahn and König, 2015) with permission of Koninklijke Brill NV.

We first calculated the committed errors as the city block distance (a distance measure also known as “Manhatten distance”) between the correct location and the selected location (see Figure 4A for a descriptive overview). We then compared the errors committed in the VIAU condition with those in the AU and VI conditions and the committed errors in the VIAU+MOT condition with those in the AU+MOT and VI+MOT conditions. For these comparisons, we used a linear model with a dummy coding scheme, with the VIAU condition and VIAU+MOT as the reference group, respectively. We found that participants committed fewer errors in the VIAU condition than in the AU or VI condition and they also committed fewer errors in the VIAU+MOT condition than in the AU+MOT condition and VI+MOT conditions (see the first and second columns in Table 2). We ran the same model with the standard deviation of the location estimates for each participant as dependent variable and found the same pattern of results: Participants' estimates of the location were less variable in the VIAU condition than in the AU and VI conditions and were also less variable in the VIAU+MOT condition than in the AU+MOT and VI+MOT conditions (see the third and fourth columns in Table 2). Overall, the findings indicate that irrespective of attentional load, participants integrate the information they receive via the visual and auditory modalities. Moreover, this pattern of results match the results in our previous study, in which participants received tactile instead of auditory spatial cues. In particular, in the previous study, we found better location estimates when receiving redundant visual and tactile spatial cues than when receiving unimodal spatial cues (for comparison, see Figure 4B), suggesting that visuotactile integration is not disrupted by attentional load.

**Table 2:** **Linear mixed model results**.

	**City block Single**	**City block Dual**	**SD Single**	**SD Dual**	**RT Single**	**RT Dual**
Intercept	0.35^*^	0.52^*^	0.26^*^	0.38^*^	0.81^*^	0.84^*^
	[0.10; 0.59]	[0.24; 0.77]	[0.17; 0.36]	[0.29; 0.47]	[0.70; 0.92]	[0.71; 0.95]
VIAU vs. AU	0.59^*^	0.54^*^	0.24^*^	0.17^*^	0.04	0.02
	[0.42; 0.76]	[0.36; 0.75]	[0.11; 0.36]	[0.07; 0.29]	[−0.02; 0.11]	[−0.05; 0.09]
VIAU vs. VI	0.50^*^	0.39^*^	0.36^*^	0.18^*^	0.07^*^	0.05
	[0.33; 0.67]	[0.19; 0.60]	[0.23; 0.47]	[0.07; 0.29]	[0.01; 0.13]	[−0.01; 0.12]
Log Likelihood	−5.94	−8.72	10.34	10.31	15.90	14.62
Participants	9	9	9	9	9	9

*Dependent variables are the city block distance (as a measurement of localization error; columns one and two for comparisons in the single task and dual task conditions), the standard deviation (“SD”) of the city block distance for each participant (columns three and four), and reaction times (“RT;” columns five and six). Unstandardized coefficient estimates of the differences between conditions are displayed. The intercept represents the mean of values in the VIAU condition, which was tested against zero (with zero representing a perfect performance for the city block error measure)*.

* *0 outside of 95% confidence interval*.

We also investigated whether the higher accuracy in the VIAU and VIAU+MOT conditions could be due to an accuracy/speed tradeoff. We tested whether the participants in the VIAU condition took longer to respond than in the AU and VI conditions and whether participants in the VIAU+MOT condition took longer to respond than in the AU+MOT and VI+MOT conditions. We did not find any significant differences between conditions (see the fifth and sixth columns in Table 2). Overall, this indicates that the better accuracy performances in the VIAU and VIAU+MOT conditions were not due to an accuracy/speed tradeoff.

## 4. Discussion

### 4.1. Do audition and vision share spatial attentional resources?

We investigated whether the auditory and visual modality share spatial attentional resources in tasks requiring sustained attention or whether there are distinct spatial attentional resources for these sensory modalities. In order to address this question, participants were asked to perform two spatial tasks (a MOT task and a LOC task) either simultaneously (dual task condition) or separately (single task conditions). Both the MOT and LOC tasks required sustained attention. We found a substantial decrease in performance in the dual task conditions relative to the single task conditions. However, we found that the amount of interference was not affected by the sensory modality in which the location cues were provided in the localization condition. That is, whether the location cues were provided via the visual, auditory, or visual and auditory modality did not affect how well participants performed the dual task. We interpret these results as an indication that shared spatial attentional resources for the auditory and visual modalities have been found for tasks requiring sustained attention. Moreover, these results are in line with results obtained in a previous study (Wahn and König, 2015), in which tactile instead of auditory spatial cues were used, suggesting that visual spatial attentional resources are shared with tactile and auditory spatial attentional resources.

However, we also want to point out that the interference between tasks in the dual task condition caused a higher performance decrease in the MOT task than in the LOC task. Given the assumption that the MOT task and LOC tasks draw from a common pool of spatial attentional resources, we would have expected that the performance decrease for both tasks would be symmetrical. We suspect that this asymmetric pattern of results can be in part explained by how the performance for each of these tasks is computed. In particular, for the MOT task and given that participants tracked three targets, misclassifying one of the targets resulted in a substantial performance decrease of one third. In contrast, in the LOC task, given that four to five location cues were received per trial, misclassifying a location cue resulted in a performance decrease of only one-fourth or one-fifth. Therefore, if participants make an equal number of mistakes in both tasks due to the interference between tasks, a performance asymmetry would still be found. In a future study, matching the number of tracked targets with the received locations cues could decrease the asymmetry of the interference.

Alternatively, this asymmetric pattern of results could be explained by an additional interference induced by the LOC task. In particular, we suspect that the executive demand of having to continuously perform key presses in the LOC task could have caused an additional interference in the MOT task that is independent of the sensory modality in which the LOC task was performed. While this alternative explanation cannot be refuted in the present study, we want to point out that finding not only a decrease in performance in the MOT task but also a decrease in performance in the LOC task for each type of location cue suggests that these two tasks indeed draw from a common pool of spatial attentional resources.

In addition, we also want to point out that participants had a better performance in the LOC task when receiving visual location cues than when receiving auditory location cues. It could be that differences in task difficulty (as indicated by localization performance) could result in different amounts of interference between tasks, independent of the sensory modalities in which the tasks are carried out. We statistically controlled for differences in localization performance to rule out any additional interference between tasks caused by differences in task difficulty. With this procedure, we assumed that there is a linear relationship between task difficulty and the interference between tasks, and this was supported by finding a trend toward significance for the relation between localization performance and task interference. After controlling for the task difficulty, we still did not find any differences between conditions, suggesting shared attentional resources between the visual and auditory modality. However, we want to point out that other (non-linear) relationships between task difficulty and interference were not controlled for and could still influence the results. In a future study, the localization performance for the auditory and visual LOC tasks could be more closely matched to circumvent the need to control for the task difficulty.

A previous study (Arrighi et al., 2011) has shown that distinct attentional resources are used for tasks requiring sustained attention. In particular, in Arrighi et al. (2011), participants were required to perform a visual spatial task (i.e., a MOT task) in combination with either a visual or auditory discrimination task, and results indicated distinct attentional resources for the visual and auditory sensory modalities. We argue that this effect may be specific to the combination of the type of tasks that were performed (i.e., a spatial task in combination with a discrimination task). In the present study, participants performed two spatial tasks instead of one discrimination task and one spatial task; we found evidence for shared attentional resources for the visual and auditory sensory modalities. Similarly, in our previous study (Wahn and König, 2015), we found indications for shared attentional resources for the visual and tactile modalities. Taken together, these findings indicate that distinct attentional resources for the sensory modalities are employed during simultaneous performance of a discrimination task and a spatial task, bit that a common pool of attentional resources is used during simultaneous performance of two spatial tasks (for a similar claim that spatial attention acts supramodally, see LaBerge, 1995; Chan and Newell, 2008).

More speculatively, we reason that our findings may be explained in terms of an overlap of neuronal populations that process both visual and auditory spatial information. Previous studies have found evidence for the existence of a dorsal “where” pathway residing in the parietal lobe, specialized in the processing of visual spatial information (Livingstone and Hubel, 1988). For the auditory modality, previous research has also found evidence for the existence of a “where” pathway, specialized in the processing of auditory spatial information and residing in the parietal lobe (Maeder et al., 2001; Ahveninen et al., 2006; and for the tactile modality see Reed et al., 2005). However, recent studies investigating the medial temporal lobe of awake monkeys provide evidence for a spatial coding of the visual location of visual overt and covert attention in these regions (Killian et al., 2012; Wilming et al., 2015). Furthermore, there are indications that separate modality-specific spatial processing systems converge at the temporoparietal junction (Coren et al., 2004). Overall, there is reason to believe that the spatial processing of stimuli from the auditory and visual modalities involves partly overlapping neuronal populations and that this could explain our finding that spatial attentional resources for the visual and auditory modalities are shared. Moreover, given that we also found shared spatial attentional resources between the visual and tactile modalities (Wahn and König, 2015), this suggests that in a future study, shared spatial attentional resources between the auditory and tactile modalities could be found as well.

In contrast, with respect to the neural correlates for a “what” pathway, specializing in object identification, previous studies indicate separate neural substrates for the visual, auditory and tactile modalities (Reed et al., 2005; Ahveninen et al., 2006; Kietzmann et al., 2012, 2013; but also see Amedi et al. (2001) for visuo-haptic object-related activation in the visual “what” pathway). These findings suggest that when two discrimination tasks are performed in separate sensory modalities, neural populations involved in processing should overlap less and evidence for distinct attentional resources could be found (see Alais et al., 2006 for distinct attentional resources when two discrimination tasks are performed; but also see Chan and Newell, 2008 for interference between two discrimination tasks performed in separate sensory modalities).

### 4.2. Is audiovisual integration disrupted by attentional load?

In addition to investigating whether spatial attentional resources are shared between the auditory and visual modalities, we also investigated whether attentional load severely interferes with multisensory integration processes. In particular, we tested whether the predictions given by multisensory cue integration were still fulfilled if participants experienced a high attentional load. Participants performed a LOC task in which they received redundant visual and auditory location cues, performing this task either alone or in combination with a MOT task. We found that, irrespective of attentional load, participants integrated the multisensory cues, as their estimates of the locations were more accurate and less variable than in unimodal conditions in which they only received either auditory or visual location cues. In contrast to previous research that has shown that audiovisual integration is susceptible to attentional load during the perception of speech (Alsius et al., 2005), our findings indicate that audiovisual integration is not disrupted by attentional load when non-speech stimuli are received which supports the view that audiovisual integration is a pre-attentive process. These findings are also in line with our previous study (Wahn and König, 2015), in which redundant visual and tactile location cues were received and integrated despite attentional load, suggesting that neither audiovisual nor visuotactile integration of spatial information is disrupted by attentional load.

However, an alternative account of our findings would be that the improved estimates of the locations when receiving multisensory location cues could be due to a trial-by-trial strategy: Participants could always use the location cue of whichever sensory modality they can interpret more accurately. Given that participants were considerably better in their location estimates when they received redundant visual and auditory location cues (rather than in comparison to only receiving unimodal cues), such an alternation account seems unlikely but cannot be fully excluded.

Another alternative explanation of our findings that multisensory integration processes were not disrupted by an additional spatial attentional load (due to simultaneous performance of a MOT task) could be that spatial attentional resources for the two different tasks are not shared. However, we believe that finding a bidirectional performance decrease in the dual task conditions suggests that the required spatial attentional resources for these two tasks indeed overlap. Therefore, we infer that the absence of a disruption of multisensory integration in the dual task could be explained by an early pre-attentive account of multisensory integration.

A possible reason that we find no effect of attentional load on audiovisual integration could be that the attentional load manipulation was not strong enough. Therefore, we cannot exclude the possibility that an even higher attentional load could lead to a disruption of audiovisual integration processes. However, given that participants had a performance of about 70 percent in the MOT task, we think that the difficulty of the MOT task was within a reasonable range that allowed for the quantification of interference between tasks without approaching floor effects.

Another possible reason that we find no affect of attentional load, in contrast to other studies (Alsius et al., 2005; Talsma et al., 2007; Mozolic et al., 2008), is that we attribute this difference in findings to the type of stimuli used. While previous studies have investigated the effect of attentional load during the perception of linguistic stimuli, the present study investigated the integration of auditory and visual location cues that did not carry any linguistic content. We suggest that the perception of linguistic stimuli could recruit additional top-down directed circuits that affect the integration of auditory and visual stimuli, which were not recruited in the present study. A future study using neurophysiological methods could contrast the use of linguistic stimuli in comparison to pure spatial information during audiovisual integration and identify the involved brain regions in conditions of high additional attentional load.

Finally, as an alternative approach for future studies, the question of whether attentional load does affect audiovisual integration could be investigated with a crossmodal congruency task (Spence et al., 2004; Walton and Spence, 2004) or a multisensory pattern matching task (Göschl et al., 2014, 2015). With such tasks, the susceptibility to distractor stimuli could be investigated as a function of attentional load and more subtle effects of attentional load may then be detected.

### 4.3. Conclusion

Our investigation of the relation between attention and multisensory processes has indicated that the type of tasks that are performed in separate sensory modalities determines whether separate attentional resources or one supramodal pool of resources is employed. Moreover, these findings suggest that the distribution of attentional resources is operating at a task level independent of the involved sensory modalities. In addition, our findings indicate that high attentional load does not disrupt the integration of spatial information from several sensory modalities, suggesting an early account of multisensory integration that is independent of attentional resources. Taken together, the findings indicate that in circumstances in which several spatial tasks need to be performed simultaneously in several sensory modalities, multisensory processes seem to operate independently from and prior to attentional processes. Future studies using a combination of EEG and fMRI could further elucidate the exact time course when multisensory and attentional processes operate, which brain regions are involved in these processes, and to what extent they operate independently or overlap in processing.

## Funding

We gratefully acknowledge the support by H2020—H2020-FETPROACT-2014 641321—socSMCs (for BW), FP7-ICT-270212—eSMCs (for PK and BW), and ERC-2010-AdG #269716—MULTISENSE (for PK).

### Conflict of interest statement

The authors declare that the research was conducted in the absence of any commercial or financial relationships that could be construed as a potential conflict of interest.
